# Electrohydrodynamic Direct-Writing Micropatterns with Assisted Airflow

**DOI:** 10.3390/mi9090456

**Published:** 2018-09-11

**Authors:** Jiaxin Jiang, Xiang Wang, Wenwang Li, Juan Liu, Yifang Liu, Gaofeng Zheng

**Affiliations:** 1 Department of Instrumental and Electrical Engineering, Xiamen University, Xiamen 361102, China; jiangjx@xmu.edu.cn (J.J.); cecyliu@xmu.edu.cn (J.L.); yfliu@xmu.edu.cn (Y.L.); 2 School of Mechanical and Automotive Engineering, Xiamen University of Technology, Xiamen 361024, China; wx@xmut.edu.cn (X.W.); xmlww@xmut.edu.cn (W.L.)

**Keywords:** electrohydrodynamic direct-writing, assisted airflow, jet stability, deposition accuracy

## Abstract

Electrohydrodynamic direct-writing (EDW) is a developing technology for high-resolution printing. How to decrease the line width and improve the deposition accuracy of direct-written patterns has been the key to the promotion for the further application of EDW. In this paper, an airflow-assisted spinneret for electrohydrodynamic direct-writing was designed. An assisted laminar airflow was introduced to the EDW process, which provided an additional stretching and constraining force on the jet to reduce the surrounding interferences and enhance jet stability. The flow field and the electric field around the spinneret were simulated to direct the structure design of the airflow-assisted spinneret. Then, a series of experiments were conducted, and the results verified the spinneret design and demonstrated a stable ejection of jet in the EDW process. With assisted airflow, the uniformity of printed patterns and the deposition position accuracy of a charged jet can be improved. Complex patterns with positioning errors of less than 5% have been printed and characterized, which provide an effective way to promote the integration of micro/nanosystems.

## 1. Introduction

Electrohydrodynamic direct-writing (EDW) [1,2] is a novel inkjet printing method, which has demonstrated the feasibility of utilizing an electric field to print micro/nanopatterns from various functional materials. By reducing the spinneret–substrate distance, organized micro/nanopatterns with an accurate positioning control can be achieved, which is not possible with other electrohydrodynamic methods, such as conventional electrospinning, which can be only used to obtain randomly distributed fibers [3,4]. Due to the advantages of low cost, simple process, and good material compatibility, the technology of EDW has presented good prospects in many fields, such as microfluidic channels [5], sensors [6], tissue engineering [7], optical devices [8], and micro/nanosystems [9].

Under the stretching of collector speed, an EDW jet can be deposited to print orderly straight lines, which has attracted lots of interests from researchers [10,11,12,13]. Bu et al. [14] reported the method of mechano-electrospinning (MES) with the help of a mechanical drawing force for high-speed collectors to direct-write oriented nanofiber with high deposition accuracy. Coppola et al. [15] invented a novel method of pyro-electrodynamic printing, introducing a wireless thermal source to activate the ferroelectric crystal, through which a pyroelectric charge was generated and fiber could be drawn out from the liquid droplet within a short working distance. Pyro-electrodynamic printing provided an excellent way to print micro/nanopatterns with a high resolution in a short time.

Due to the surrounding interferences and repulsive force from the residual charge on the deposited structures generated by the high electrical field during the EDW process [16,17], it is easy for the jet to step into an instability stage when the distance between spinneret and collector is short, which hampers the high-resolution deposition of EDW patterns. In general electrohydrodynamic applications, there is usually a high printing speed to obtain straight lines [18,19,20,21], which would nevertheless result in a large deviated angle of the charged jet, making it difficult to print complex patterns in microscales. The relative motion of the spinneret and the collector is the key factor for the precise deposition of micro/nanopatterns, which is of great importance in the field of micro/nanosystem integration manufacturing, such as sensor systems [22], electronic components [23], metal electrodes [24], and organic nanowires [25]. Therefore, in recent years, several methods have been proposed to promote the ejection stability and deposition precision of charged jets to direct-write complex patterns. Nicolas et al. [26] used an auxiliary electrode to generate a secondary electric field to guide the jet flight in a straight trajectory, so that the position accuracy of deposited structures was increased. Lee et al. [27] introduced a focused sharp-pin electrode into the EDW process to fabricate various patterns with high geometric fidelity. Luo et al. [28] utilized printing paper as the collector to accelerate the charge transferred to ground by infiltrating the residual solvent into substrate, resulting in several complex logos. It has been reported that the focused airflow contributes to the shielding of interferences and the promotion of viscous microjet stability [29,30,31]. With the external stretching force, the line width of direct-written patterns is decreased and the voltage for jet ejection is reduced; thus, the charge density on the jet is decreased and jet stability is enhanced during the EDW process [32]. In addition, the airflow constrains the jet in a straight flight, which decreases the requirement for a high printing speed to realize the controllable position, which would promote the application of EDW with simple controlling systems. However, further discussion on the interaction between the focused airflow and the charged jet is still required.

In this paper, an airflow-assisted spinneret is designed to optimize the EDW process. Simulation and experimental results demonstrate that assisted airflow provides an effective way to promote jet ejection and patterned deposition during the EDW process.

## 2. Materials and Methods

**Figure 1:**
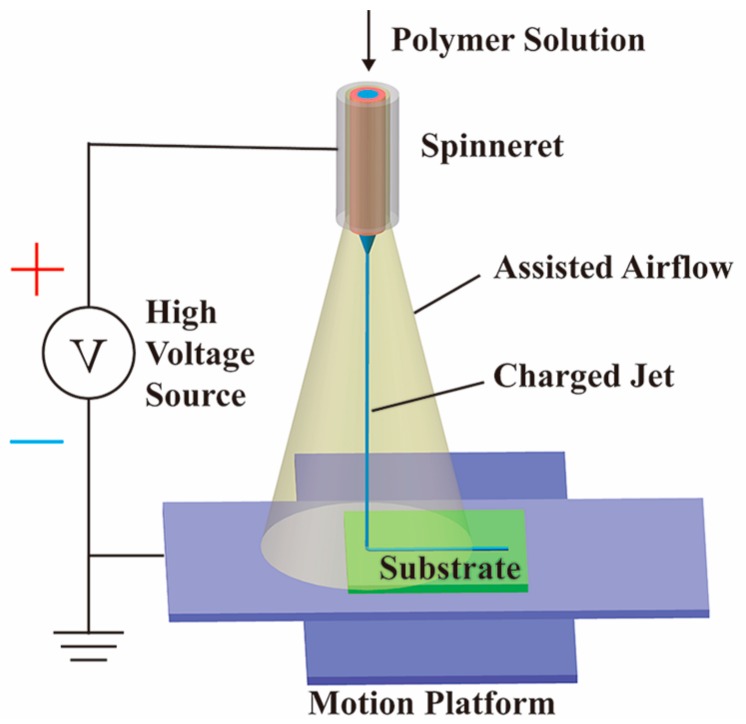
Schematic diagram of electrohydrodynamic direct-writing (EDW) with assisted airflow.

The schematic diagram of EDW with assisted airflow is illustrated in Figure 1. The anode of the high-voltage source (DW-SA403-1ACE5, Dongwen high-voltage power source Ltd. of Tianjin, Tianjin, China) was connected to the spinneret, including an outer airflow passage with a diameter of 1 mm and an inner liquid passage with a diameter of 0.21 mm, the two passages of which were arranged coaxially. The cathode was connected to the silicon substrate, which was fixed on an XY motion platform (GXY1515GT4, Googoltech, Shenzhen, China); thus, a high electric field was generated between the spinneret and the grounded substrate. The silicon substrate was ultrasonically cleaned in acetone and dried in the air flow before the experiments. In the experiments, the distance between the tip of the spinneret and the substrate was fixed to be 2 mm. Under the stretching of a high electric field within a short distance, a straight charged jet could be ejected from the tip of Taylor Cone and deposited into a controllable pattern on the substrate.

A polyethylene oxide (PEO, molecular weight = 300,000 g/mol) aqueous solution with a concentration of 8 wt % was used as the EDW liquid, the solvent of which was formed by mixing the deionized water and the ethanol with a volume ratio of 3:1. Then, the solution was supplied into the liquid passage of the spinneret with a precision syringe pump (Pump 11 Pico Plus Elite, Harvard Apparatus, Holliston, MA, USA) at a constant flow rate of 30 μL/h, and the airflow was provided by a gas pump at a constant pressure of 25 kPa. During the experiments, a charge-coupled device (CCD) camera (Sony SSC-DC80, Sony Corporation, Tokyo, Japan) was used to record the ejecting behavior of a charged jet, and an optical microscope (Mitutoyo, Kawasaki, Japan) was used to observe the morphology of direct-written patterns. The average width was calculated from more than 50 data points in 10 samples, which were analyzed by the ImageJ software.

**Figure 2:**
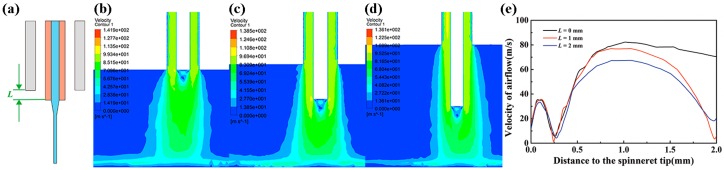
Flow field simulation for different spinneret designs. (**a**) Schematic diagram for the airflow-assisted spinneret; (**b**) flow field simulation when *L* = 0 mm; (**c**) flow field simulation when *L* = 1 mm; (**d**) flow field simulation when *L* = 2 mm; (**e**) distribution of airflow velocity for different spinneret designs.

**Figure 3:**
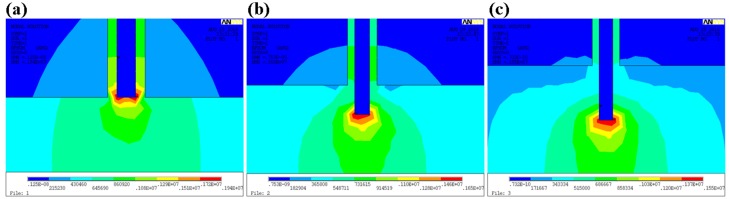
Electrical field simulation for different spinneret designs. (**a**) *L* = 0 mm; (**b**) *L* = 1 mm; (**c**) *L* = 2 mm.

To optimize jet behavior from the airflow-assisted spinneret, it was found that one major parameter was the vertical distance between the gas outlet and the liquid outlet (*L*), as presented in Figure 2a. The airflow distribution and velocity between the spinneret and substrate was simulated for different configurations, as shown in Figure 2b–d. The results indicated that a small *L* led to a turbulent flow above the substrate, which would aggravate the disturbance of the charged jet. However, when *L* was elongated to a certain range, the airflow above the substrate would be in the laminar flow, and the charged jet would suffer an additional stretching and constraining force, as shown in Figure 2e, which was beneficial for the promotion of jet stability. Electrical field distribution was also an important factor affecting jet-ejecting behavior, as simulated in Figure 3 for different spinneret designs. When *L* = 0 mm, there was a divergent electric field around the spinneret, making it hard to stretch the jet just in a vertical direction. However, when the liquid outlet was below the gas outlet, the electric field lines were mostly concentrated below the tip of the spinneret, which could help to reduce the voltage for jet ejection and the instability for the motion behavior of a charged jet.

## 3. Results and Discussion

**Figure 4:**
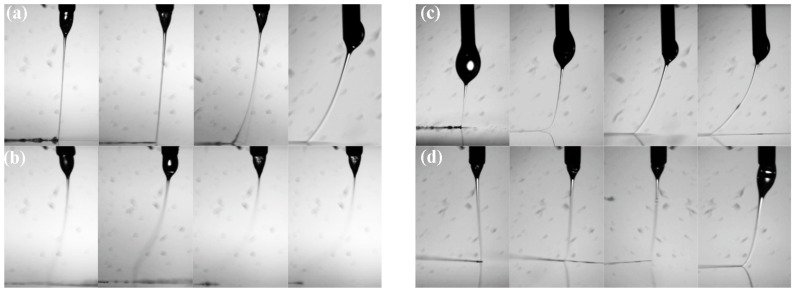
The behavior of charged jets under different spinneret configurations when the collector moving speed was 1 mm/s, 5 mm/s, 10 mm/s, and 20 mm/s, respectively. (**a**) Without assisted airflow when *L* = 0.5 mm, the applied voltage was 2.3 kV; (**b**) with assisted airflow of 25 kPa when *L* = 0.5 mm, the applied voltage was 2.3 kV; (**c**) without assisted airflow when *L* = 2 mm, the applied voltage was 2.0 kV; (**d**) with assisted airflow of 25 kPa when *L* = 2 mm, the applied voltage was 2.0 kV.

**Figure 5:**
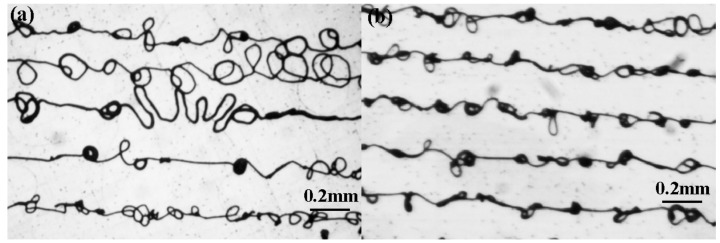
Direct-written parallel lines with assisted airflow of 25 kPa. (**a**) *L* = 0.5 mm; (**b**) *L* = 2 mm. The collector moving speed was 1 mm/s.

Firstly, jet behaviors with different spinneret designs are investigated in Figure 4. Under the high electric field force, the droplet at the outlet of liquid passage was stretched into a Taylor Cone, and a fine charged jet was ejected from the tip of liquid cone. When *L* = 0.5 mm, the smallest applied voltage was 2.3 kV to maintain the continuous ejection of jet (Figure 4a), while when *L* = 2 mm, it was reduced to 2.0 kV (Figure 4c), which verified that a longer *L* was beneficial for a concentrated electrical field, consistent with Figure 3. Without the help of assisted airflow, the charged jet was deviated from the vertical line under the stretching of a moving collector. When the speed of the moving collector increased, the deviated distance became larger, which hindered the accurate deposition of a charged jet. However, with the constraint of assisted airflow, the deviated distance was largely decreased and the charged jet could be maintained on a straight line track when the collector moving speed was lower than 10 mm/s when *L* = 2 mm, as shown in Figure 4d. While *L* = 0.5 mm, the jet was much more unstable than that without airflow, as shown in Figure 4b, due to the disturbance from a turbulent flow below the spinneret, presented in Figure 2. The corresponding direct-written lines with an assisted airflow of 25 kPa are given in Figure 5. It can be seen that the deposited lines with *L* = 2 mm had fewer whipping structures than those with *L* = 0.5 mm, which is attributed to the laminar flow of assisted airflow below the spinneret, which constrained the deposition position of the charged jet precisely. Therefore, *L* = 2 mm was utilized in the following experiments to specify the effects of assisted airflow.

**Figure 6:**
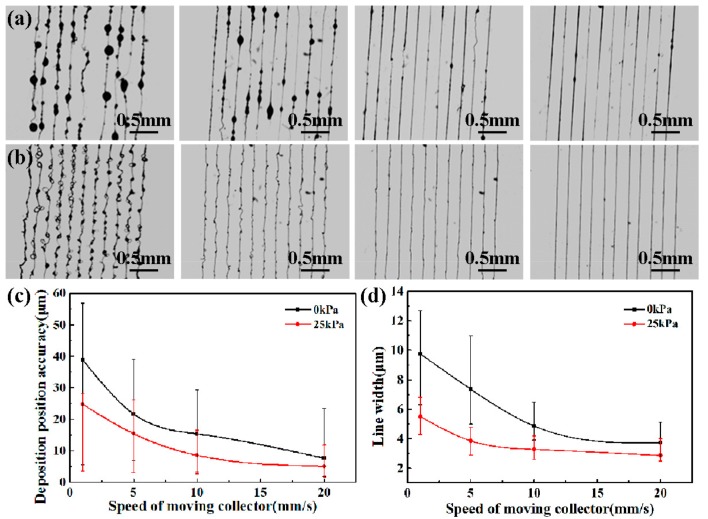
Direct-written parallel lines when collector moving speed was 1 mm/s, 5 mm/s, 10 mm/s, 20 mm/s, respectively. (**a**) Without assisted airflow; (**b**) with assisted airflow of 25 kPa; (**c**) relationship between deposition position accuracy and collector moving speed; (**d**) relationship between line width and collector moving speed. The applied voltage was 2.0 kV.

**Figure 7:**
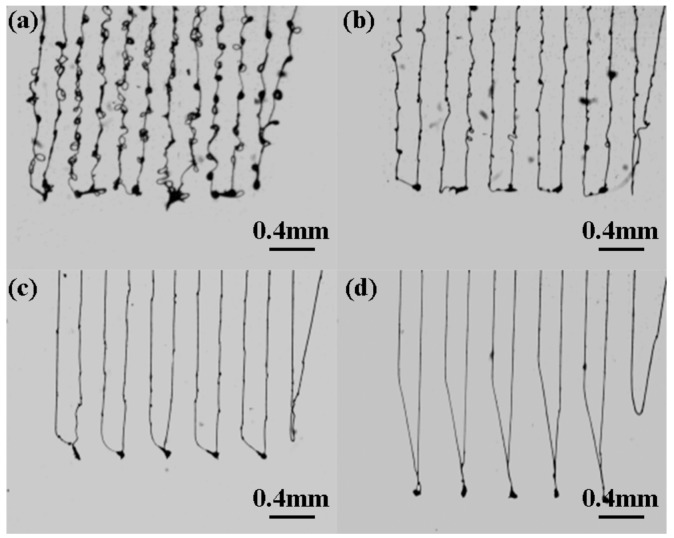
Direct-written patterns at the turning point under collector moving speed. (**a**) 1 mm/s; (**b**) 5 mm/s; (**c**) 10 mm/s; (**d**) 20 mm/s. The assisted airflow and the applied voltage were 25 kPa, 2.0 kV, respectively.

The parallel lines were direct-written under a different collector moving speed, as shown in Figure 6. From the results, it could be seen that the deposition position accuracy was improved and the line width was decreased with the constraint of airflow. The assisted airflow promoted the evaporation of solvent and the solidification of micro/nanostructures; thus, the bead structures in the deposited lines disappeared with an assisted airflow of 25 kPa, as shown in Figure 6b. In addition, collector moving speed was the main factor determining the morphology of printed patterns [2]. When collector moving speed was lower than 10 mm/s, wavy patterned structures were formed, rather than a straight line. While collector moving speed was approximately to 10 mm/s, the charged jet could be deposited into a straight line structure without spiral motion, and the direct-written patterns could be achieved well. However, when the collector moving speed was increased further, there was a significant deviated distance from the vertical line of the charged jet, as shown in Figure 3. It was also difficult to obtain a correct pattern at the turning point due to the fast change of the collector moving direction at a high speed, as shown in Figure 7.

Moreover, the effects of collector moving speed and assisted airflow on the deposition position accuracy and the line width of printed patterns are discussed as Figure 6c,d. When collector moving speed increased from 1 mm/s to 20 mm/s, the deposition position accuracy of direct-written patterns decreased from 38.9 ± 33.4 μm to 7.71 ± 15.79 μm, and 24.79 ± 21.29 μm to 5.13 ± 6.67 μm, while the line width of direct-written patterns decreased from 9.76 ± 3.46 μm to 3.73 ± 1.37 μm, and 5.51 ± 1.29 μm to 2.87 ± 1.13 μm when the assisted airflow was 0 kPa and 25 kPa, respectively.

**Figure 8:**
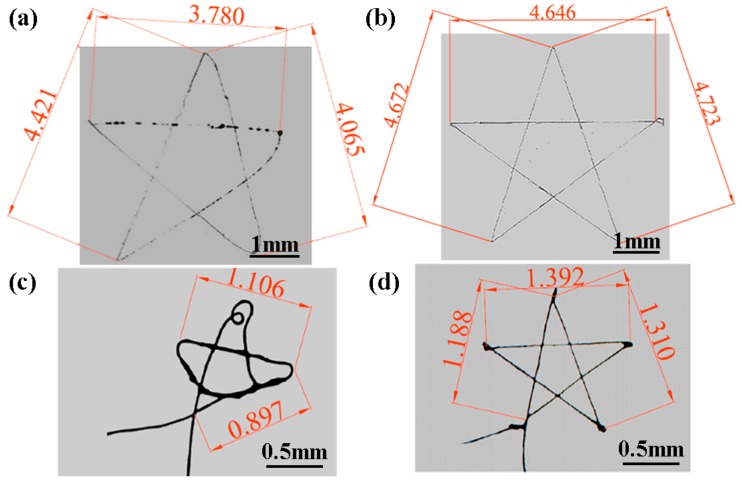
Pentagram patterns direct-written under various parameters. (**a**) Without assisted airflow when pentagram side length was 4.76 mm; (**b**) with assisted airflow of 25 kPa when pentagram side length was 4.76 mm; (**c**) without assisted airflow when pentagram side length was 1.33 mm; (**d**) with assisted airflow of 25 kPa when pentagram side length was 1.33 mm. The collector moving speed and the applied voltage were 10 mm/s, 2.0 kV, respectively.

In addition, complex pentagram patterns in different scales were achieved to demonstrate the feasibility of an airflow-assisted spinneret for high-resolution EDW, as shown in Figure 8. Without the assistance of airflow, the deposited patterns were easily affected by the residual charges on the substrate and the fast change of collector moving speed, so that it was difficult to obtain a well-patterned complex pattern with high precision. However, the assisted airflow provides a strong constraint and stretching force on the jet to weaken the interference from residual charges and surrounding conditions, resulting in a high ejecting speed of jet, so that the accurate complex pattern of a pentagram was direct-written with a positioning error of less than 5%, corresponding to the designed geometric drawing even with a total scale of less than 1.5 × 1.5 mm^2^ under a collector moving speed of 10 mm/s.

## 4. Conclusions

The laminar airflow was introduced into the process of EDW, and the structure of an airflow-assisted spinneret was optimized. Assisted airflow provided an external stretching force and constraining effect on the charged jet to maintain it on a straight track, by overcoming the interferences from residual charges and high-speed substrate. Flow field simulations were conducted to investigate the effect of vertical distance between the gas outlet and the liquid outlet to guide the structure design of the spinneret. With the assistance of airflow, the evaporation of solvent and solidification of micro/nanostructures were accumulated, and the bead structures in the direct-written patterns could be made to disappear. The effect of the collector moving speed on the printed patterns was studied as well. When collector moving speed increased from 1 mm/s to 20 mm/s, the average deposition position accuracy of direct-written patterns decreased from 24.79 μm to 5.13 μm, while the line width of direct-written patterns decreased from 5.51 μm to 2.87 μm with an assisted airflow of 25 kPa. By matching the processing parameters, complex pentagram patterns with a high deposition position accuracy were achieved. The novel airflow-assisted spinneret provides a simple way for high-resolution EDW, and is expected to promote the application of EDW in micro/nanomanufacturing.
